# Splitting the *Pisonia* birdcatcher trees: re-establishment of *Ceodes* and *Rockia* (Nyctaginaceae, Pisonieae)

**DOI:** 10.3897/phytokeys.152.50611

**Published:** 2020-07-07

**Authors:** Elson Felipe Sandoli Rossetto, Marcos A. Caraballo-Ortiz

**Affiliations:** 1 Departamento de Biologia Animal e Vegetal, Centro de Ciências Biológicas, Universidade Estadual de Londrina, Campus Universitário, Rodovia Celso Garcia Cid, PR 445, Km 380, 86057-970, Londrina, PR, Brazil Universidade Estadual de Londrina Londrina Brazil; 2 Smithsonian Institution, National Museum of Natural History, Department of Botany, MRC 166, Washington, DC 20013‐7012, USA Smithsonian Institution Washington, DC United States of America

**Keywords:** *
Calpidia
*, Caryophyllales, flora of Hawaii, flora of the Indo-Pacific, flora of oceanic islands, *
Heimerliodendron
*, island endemics, *
Timeroyea
*

## Abstract

Several genera of Nyctaginaceae, currently merged under *Pisonia*, have been described for the Indo-Pacific region. Results from a recent molecular phylogenetic study of tribe Pisonieae showed that *Pisonia* is non-monophyletic and comprises three well-supported lineages: one including typical *Pisonia* and allies (*Pisonia* s.str.), a clade of species which corresponds to the original description of *Ceodes* and a third lineage whose single representative was formerly treated under the monotypic genus *Rockia*. Thus, as part of an effort to achieve a natural classification for tribe Pisonieae, this work proposes to re-establish *Ceodes* and *Rockia* to accommodate taxa with inconspicuous glands on anthocarps, recognising 21 species (20 for the former and one for the latter), of which 16 are new combinations: *Ceodes amplifolia***comb. nov.**, *Ceodes artensis***comb. nov.**, *Ceodes austro-orientalis***comb. nov.**, *Ceodes brownii***comb. nov.**, *Ceodes cauliflora***comb. nov.**, *Ceodes coronata***comb. nov.**, *Ceodes diandra***comb. nov.**, *Ceodes gigantocarpa***comb. nov.**, *Ceodes gracilescens***comb. nov.**, *Ceodes lanceolata***comb. nov.**, *Ceodes merytifolia***comb. nov.**, *Ceodes muelleriana***comb. nov.**, *Ceodes rapaensis***comb. nov.**, *Ceodes sechellarum***comb. nov.**, *Ceodes taitensis***comb. nov.** and *Ceodes wagneriana***comb. nov.** A general distribution of each species recognised in this work is also included, along with line drawings and colour pictures of representative species of *Ceodes*, *Pisonia* and *Rockia* and an updated dichotomous key based on reproductive characters for the nine genera (*Ceodes*, *Cephalotomandra*, *Grajalesia*, *Guapira*, *Neea*, *Neeopsis*, *Pisonia*, *Pisoniella* and *Rockia*) comprising the tribe Pisonieae.

**Résumé**

Plusieurs genres de Nyctaginaceae actuellement fusionnés sous *Pisonia* ont été décrits pour la région Indo-Pacifique. Les résultats d’une récente étude phylogénétique moléculaire de la tribu Pisonieae ont montré que *Pisonia* est non monophylétique et comprend trois lignées bien supportées: une comprenant *Pisonia* typique et ses alliés (*Pisonia* s.str.), un clade d’espèces qui correspond à la description originale de *Ceodes* et une troisième lignée dont l’unique représentant était auparavant traité sous le genre monotypique *Rockia*. Ainsi, dans le cadre d’un effort pour parvenir à une classification naturelle de la tribu Pisonieae, ce travail proposons de rétablir les *Ceodes* et *Rockia* pour accueillir des taxons avec des glandes discrètes sur les anthocarpes, reconnaissant 21 espèces (20 pour les premières et une pour les dernières), dont 16 sont de nouvelles combinaisons: *Ceodes amplifolia***comb. nov.**, *Ceodes artensis***comb. nov.**, *Ceodes austro-orientalis***comb. nov.**, *Ceodes brownii***comb. nov.**, *Ceodes cauliflora***comb. nov.**, *Ceodes coronata***comb. nov.**, *Ceodes diandra***comb. nov.**, *Ceodes gigantocarpa***comb. nov.**, *Ceodes gracilescens***comb. nov.**, *Ceodes lanceolata***comb. nov.**, *Ceodes merytifolia***comb. nov.**, *Ceodes muelleriana***comb. nov.**, *Ceodes rapaensis***comb. nov.**, *Ceodes sechellarum***comb. nov.**, *Ceodes taitensis***comb. nov.** et *Ceodes wagneriana***comb. nov.** Une distribution générale de chaque espèce reconnue dans ce travail est également incluse, ainsi que des dessins au trait et des images en couleur des espèces représentatives de *Ceodes*, *Pisonia* et *Rockia*, et préparé une clé dichotomique mise à jour basée sur les caractères reproductifs des neuf genres (*Ceodes*, *Cephalotomandra*, *Grajalesia*, *Guapira*, *Neea*, *Neeopsis*, *Pisonia*, *Pisoniella* et *Rockia*) comprenant la tribu Pisonieae.

## Introduction

**Figure 1.**
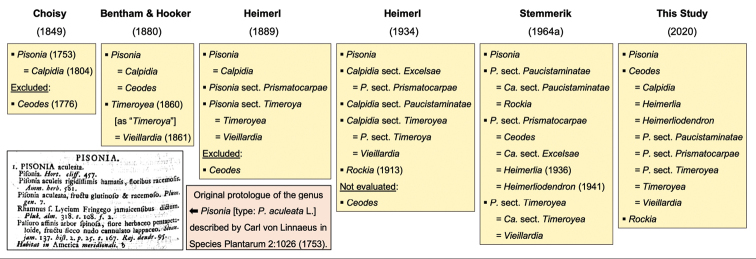
Diagram depicting the history of classification for *Calpidia*, *Ceodes*, *Heimerliodendron*, *Pisonia* and *Rockia*. Major taxonomic treatments are shown within boxes. Authors are shown in bold and publication years of treatments and genera within parentheses.

The tribe Pisonieae Meisn. in Nyctaginaceae (Caryophyllales) contains the most diverse woody assemblage of the family, represented by over 200 species distributed mainly in the tropical and subtropical regions of the New World (Douglas and Spellenberg 2010). Although members of Pisonieae are present – and often common – in all types of Neotropical habitats and are important components for many ecosystems, taxonomic delimitations at the generic and species levels are still obscure and in urgent need of updated treatments. The tribe currently comprises seven accepted genera (*Cephalotomandra* H.Karst & Triana, *Grajalesia* Miranda, *Guapira* Aubl., *Neea* Ruiz & Pav., *Neeopsis* Lundell, *Pisonia* L. and *Pisoniella* (Heimerl) Standl.), all of them restricted to the New World except *Pisonia*, which has a pantropical distribution (Douglas and Spellenberg 2010). However, other genera have been erected in the last three centuries to include some of the Indo-Pacific taxa with dried anthocarps and inconspicuous glands along anthocarp ribs, which have been either recognised as accepted or treated as synonyms of *Pisonia* by different authors, resulting in a convoluted taxonomic history that we aim to clarify below and that is also summarised in Figure 1.

**Figure 2.**
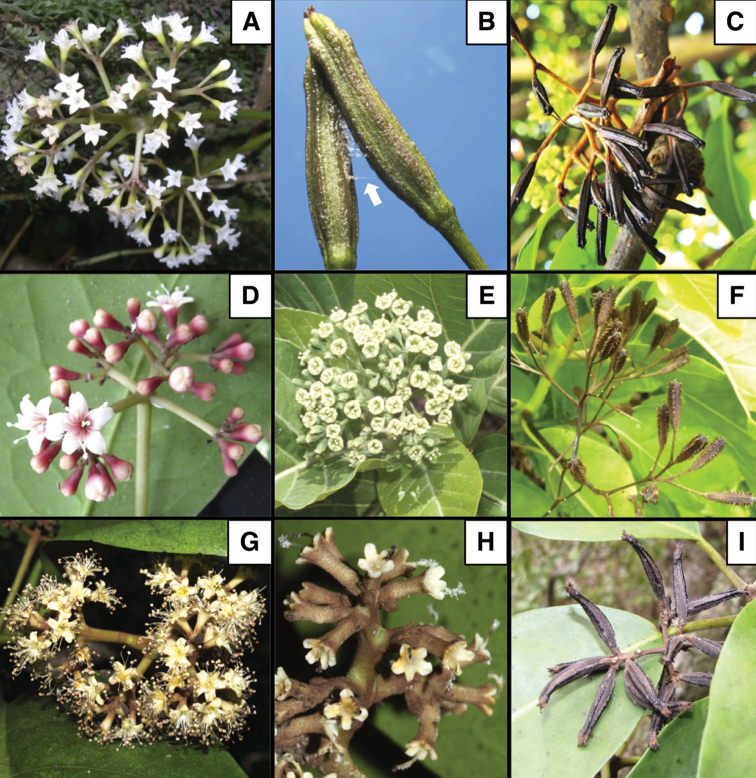
Field images for representative species of *Ceodes*, *Pisonia* and *Rockia* (Nyctaginaceae) from the Pacific Islands **A***Ceodes taitensis*. Branch with pistillate flowers **B***Ceodes brunoniana*. Ripe anthocarps (fruits) exuding sticky secretions (arrow) **C, D***Ceodes umbellifera*. Branch with ripe anthocarps (**C**) and staminate flowers at anthesis (**D**) **E, F***Pisonia grandis* R.Br. Staminate flowers at anthesis (**E**) and ripe anthocarps (**F**) **G–I***Rockia sandwicensis*. Staminate (**G**) and pistillate (**H**) flowers at anthesis and ripe anthocarps (**I**) Photo credits: **A, F** by J.-Y. Meyer **B** by L. Jensen **C** by C.-I Peng **D, E, I** by F. Starr and K. Starr **G, H** by K. Magnacca.

**Figure 3.**
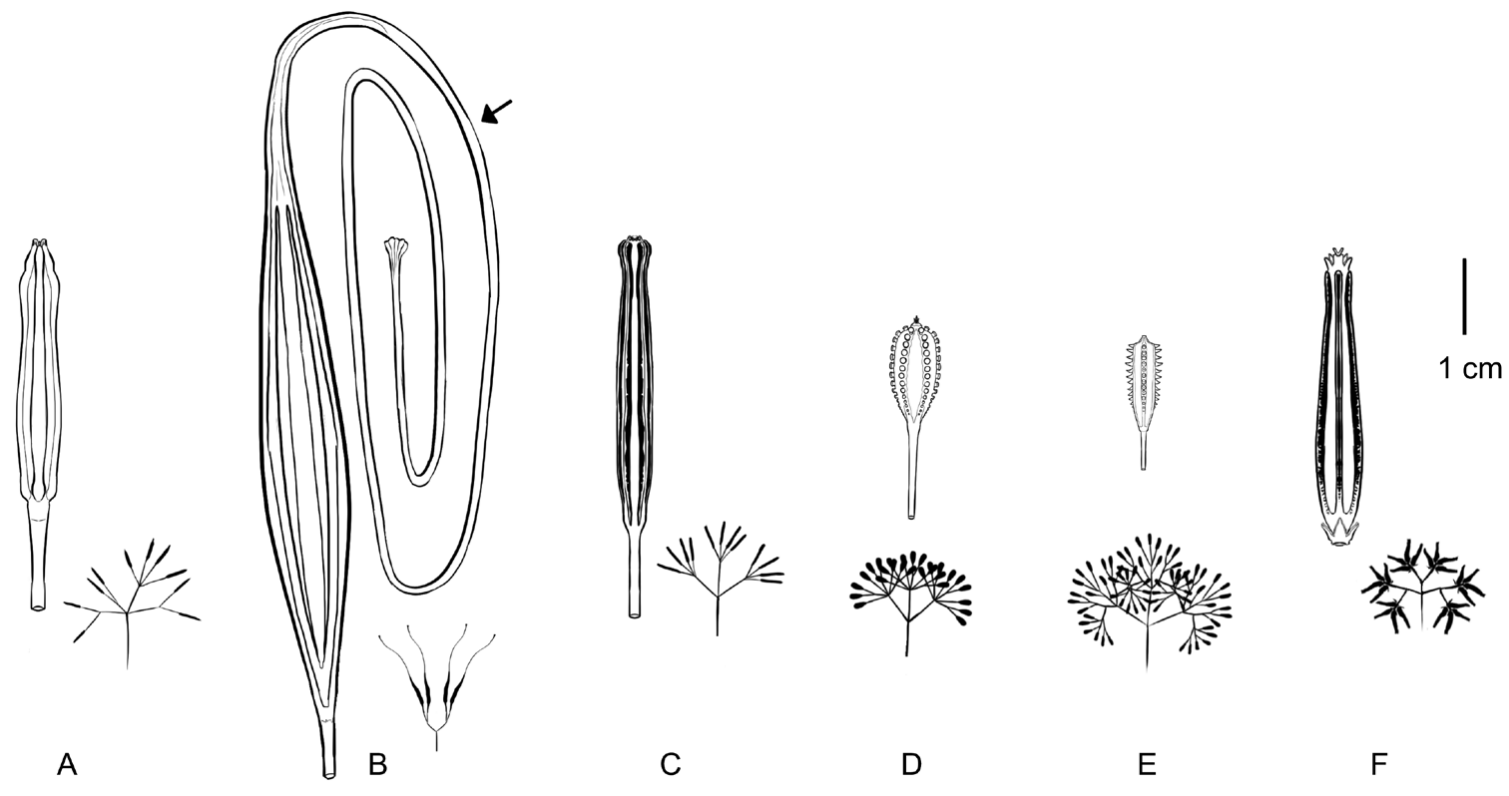
Comparison of size and morphology of ripe fruits (anthocarps) amongst members of *Ceodes*, *Pisonia* and *Rockia* (Nyctaginaceae). The outline of infructescences are shown below each anthocarp **A***Ceodes brunoniana* (based on *St. John 11272* (US-01258187)) **B***Ceodes longirostris* (based on *Brass 2972* (HUH-00046918)). Note the extremely long rostrum at the tip of the anthocarp (indicated with an arrow) **C***Ceodes umbellifera* (based on *Foxworthy 593* (US-03661041)) **D***Pisonia aculeata* L. (based on *Caraballo 3464* (IJ)) **E***Pisonia grandis* (based on *Fosberg 24357* (US-00959523)) **F***Rockia sandwicensis* (based on *Lorence 6305* (US-00452890)). Illustration credit: Ramos Sepúlveda.

*Ceodes* J.R. Forst. & G.Forst., which was described by Forster and Forster (1776), is the oldest of the Indo-Pacific genera with its type species *Ce. umbellifera* J.R.Forst. & G.Forst. collected in Tanna Island at Vanuatu, characterised by the absence of stalked glands along the ribs of anthocarps (Seemann 1863). Later, Du Petit-Thouars (1804) established the genus *Calpidia* Thouars, whose detailed description is based on material collected in Mauritius and which, just as *Ceodes*, differed from *Pisonia*, mainly by the absence of glandular emergences on the surface of its anthocarps (Du Petit-Thouars 1806; see Figs 2, 3). The protologues of *Ceodes* and *Calpidia* describe essentially the same diagnostic characters and it is possible that Du Petit-Thouars was unaware that *Ceodes* had been described 28 years earlier, as he did not mention this genus in either of his two publications on *Calpidia*.

In a global treatment of Nyctaginaceae, prepared by Choisy (1849), he merged *Calpidia* under *Pisonia* and, as with Du Petit-Thouars, he did not include *Ceodes* in this treatment. Over 30 years later, Bentham and Hooker (1880) maintained both *Calpidia* and *Ceodes* as synonyms of *Pisonia*. However, they recognised the monotypic genus *Timeroyea* Montrouz. (using the orthographic variant “*Timeroya*”) from New Caledonia, which besides *Ceodes* and *Calpidia*, represents a third genus with inconspicuous glands on its anthocarps, but was characterised by having many (25–30) stamens (Montrouzier 1860; Beauvisage 1901).

Nine years after Bentham and Hooker’s publication, Heimerl (1889) presented his first tribal treatment for Nyctaginaceae, in which he split *Pisonia* into six sections, resulting in an expanded delimitation of this genus. *Timeroyea* was reduced to a section of *Pisonia* (as P. sect. Timeroya), while P. sect. Prismatocarpae was established to accommodate taxa with up to 15 stamens, resulting in two sections of *Pisonia* with inconspicuously-glanded taxa. In this treatment, *Calpidia* was listed as a synonym of *Pisonia*, while *Ceodes* was not mentioned at all.

Subsequent work by Heimerl (1913a), based on the examination of additional material, led him to propose the split of *Pisonia* sensu lato with the reinstatement of *Calpidia* to embrace all taxa placed under P. sect. Prismatocarpae and P. sect. Timeroya. Here, *Calpidia* differed from other *Pisonia* sections by the absence of bracteoles, having a reduced perisperm that forms gelatinous traces and starch accumulation in the embryo and by its geographic distribution. In the following publication which included palynological analysis, Heimerl (1913b) proposed new combinations to *Calpidia*, including some of the newly described species by Warburg (1891) and Bargagli-Petrucci (1901) and described the monotypic genus *Rockia* Heimerl to accommodate the Hawaiian endemic *P. sandwicensis* Hillebr. Although the anthocarps of *Rockia* have inconspicuous glands located along the ribs, Heimerl (1913b) distinguished it from *Calpidia* by the presence of bracteoles and the number of pores in pollen grains, where *Rockia* has sessile flowers with one bract and two bracteoles at its base and tricolpate pollen, while *Calpidia* has pedicellate flowers lacking any bracts or bracteoles at the upper portion of the pedicels and pollen with four or more colpi, resulting in many apertures.

In his last comprehensive synthesis of Nyctaginaceae, Heimerl (1934) maintained *Rockia* as an accepted genus and additionally recognised three sections in *Calpidia*, based on the presence of a rostrum – a beaked or filamentous extension at the apex of the anthocarps – and the number of stamens: Ca. sect. Excelsae (= P. sect. Prismatocarpae), Ca. sect. Paucistaminatae and Ca. sect. Timeroyea. Calpidia sect. Timeroyea, represented by the New Caledonian endemic *Ca. artensis* (Montrouz.) Heimerl, is characterised by flowers with a high number of stamens (≥ 30), while the Indo-Pacific widespread Ca. sect. Excelsae, which includes *Ca. excelsa* (Blume) Heimerl and *Ca. brunoniana* (Endl.) Heimerl, has flowers with 6–30 stamens. On the other hand, species with less than five stamens and a rostrum were placed into Ca. sect. Paucistaminatae, most of whose representatives occur in Papua New Guinea (e.g. *Ca. longirostris* (Teijsm. & Binn.) Heimerl).

Unlike with his former treatment, Heimerl (1934) mentioned *Ceodes*, but did not evaluate its status under the argument that he lacked enough information about this genus to reach a taxonomic decision, even when Skottsberg (1926), years before, had acknowledged the priority of *Ceodes* over *Calpidia*. Skottsberg re-established *Ceodes* under the argument that there were extant original specimens and that the scant description of the genus was similar to other names published during the late 18^th^ century. However, Skottsberg’s treatment was restricted to the plants from Hawai‘i and included only two species in *Ceodes* (*Ce. brunoniana* (Endl.) Skottsb. and *Ce. forsteriana* (Endl. ex Walp.) Skottsb.) and listed a third one (*Ce. excelsa* (Blume) Skottsb.) as a questionable species. As Heimerl (1934) still did not accept *Ceodes* and retained *Calpidia* in his new treatment of Nyctaginaceae, Skottsberg (1936) published a work focused on the nyctaginaceous trees from Hawai‘i where he reinstated his views on the priority of *Ceodes*, this time recognising only one species (*Ce. umbellifera*, including *Ce. excelsa* and *Ce. forsteriana* as synonyms). In this same work, he also described the genus *Heimerlia* Skottsb. to accommodate *Ce. brunoniana*, a species characterised by having hermaphroditic flowers. Following Skottsberg’s views, Heimerl (1937) finally accepted the priority of *Ceodes* over *Calpidia* and described a new form for *Ce. umbellifera* (Ce. umbellifera f. amplifolia Heimerl), but did not effectuate any transfers from the sections of *Calpidia* he previously published. Two years later, in their first paper on a series of publications on the plants from Papua New Guinea, Merrill and Perry (1939) proposed two new combinations for species of *Ceodes*. Finally, Skottsberg (1941) corrected the name *Heimerlia* to *Heimerliodendron* Skottsb. after noticing that the former had been already described for a fungus.

In a new and drastically different treatment for the group, Stemmerik (1964a) proposed a broad definition for *Pisonia* which re-incorporated all taxa with inconspicuous glands along anthocarps. In his revision, which was restricted to the Indo-Pacific taxa, he merged *Calpidia*, *Ceodes*, *Heimerliodendron* and *Rockia* within *Pisonia*. The three sections of *Calpidia*, recognised by Heimerl in 1934, were transferred to *Pisonia*, where P. sect. Prismatocarpae sensu Heimerl (1889) (same as Ca. sect. Excelsae sensu Heimerl 1934) was restored. *Rockia* was merged into P. sect. Paucistaminatae (sensu Heimerl 1934) along with the taxa with pedicellate flowers and having an anthocarp rostrum. Therefore, the delimitation of the three sections of *Pisonia*, proposed by Stemmerik (1964a), was based on characters used by Heimerl (1934) to define his sectional ranks, such as type of glands on anthocarps, presence of a rostrum and number of stamens. However, he did not consider the absence of bracts and bracteoles at the upper portion of the pedicels, presence of starch in the embryo and number of apertures in pollen grains, as he argued that at least the pollen structure was not a constant character and, therefore, had no utility separating genera (Stemmerik 1964b).

**Figure 4.**
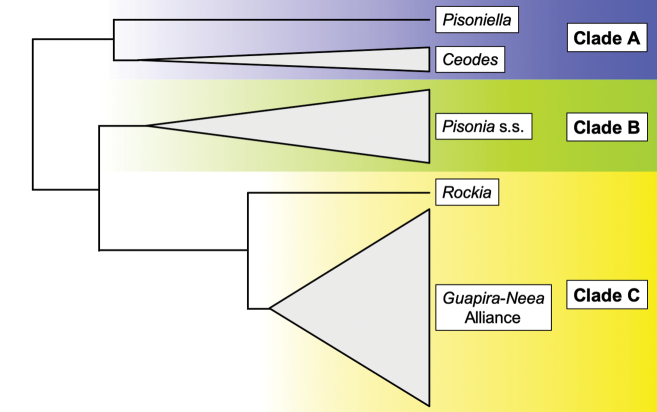
Generic relationships within tribe Pisonieae (Nyctaginaceae) showing the inferred positions of *Ceodes* (Clade A, in blue), *Pisonia* (Clade B, in green) and *Rockia* (Clade C, in yellow) (Rossetto et al. 2019).

A recent phylogenetic study of tribe Pisonieae, based on molecular data (Rossetto et al. 2019), indicated that *Pisonia*, as delimitated by Stemmerik (1964a, b), is non-monophyletic. The current definition of *Pisonia* places taxa with inconspicuous glands along anthocarp ribs (Fig. 3A–C, F) into two distinct, well-supported lineages (i.e. clades A and C sensu Rossetto et al. 2019; Fig. 4), while typical *Pisonia* and its allies with glandular emergences (Fig. 3D–E) are restricted to clade B (*Pisonia* s.str.; Fig. 4). In clade A, formed by taxa carrying pedicellate flowers without bracteoles, members of the P. sect. Prismatocarpae and P. sect. Timeroyea (sensu Stemmerik 1964a) are included in the clade Ceodes, although these sections were not clustered in natural groups. Concurrently, *P. sandwicensis* from the P. sect. Paucistaminatae is placed in clade C as sister to the Neotropical genera *Guapira* and *Neea* (Rossetto et al. 2019; Fig. 4). Therefore, in order to simplify the classification of the tribe by designating monophyletic genera for the two independent lineages with inconspicuous glands within the tribe, it is necessary to resurrect *Ceodes* and *Rockia*. The objective of this work is to re-establish these two genera and to provide new combinations where necessary. To facilitate recognition in herbaria and in the field, we also provide colour pictures and line drawings of representative species of *Ceodes*, *Pisonia* and *Rockia* and a dichotomous key for the nine genera comprising the tribe Pisonieae.

## Materials and methods

For the taxonomic treatment, we compiled accepted names following Stemmerik’s revision (1964a), which is the most recent comprehensive treatment for Pisonieae in the Pacific region. We also consulted other more recent regional treatments and species descriptions and provided new generic combinations of the taxa that, according to our understanding, are currently considered as accepted (Friedmann 1986; Fosberg 1987; Philcox and Coode 1994; Florence 2004; Whistler 2004). Additional information on geographic distributions was obtained from Heimerl (1913b) and Stemmerik (1964b). Generic descriptions were based on Heimerl (1913b; 1934), while Skottsberg (1936) was used specifically for the description of pollen structure of *Ceodes*.

## Results and conclusions

Here we re-established the genera *Ceodes* and *Rockia*, recognising 20 species for the former and one for the latter. Sixteen out of the 20 species, recognised for *Ceodes*, represent new combinations (see Taxonomic treatment section). The re-establishment of *Ceodes* and *Rockia* provides an important step to refine our knowledge of the taxonomy and evolution of Pisonieae from the Indo-Pacific region. This work also has considerable implications for estimates of regional biodiversity, as many species of *Ceodes* are island endemics, while *Rockia* would be a genus restricted to the Hawaiian Archipelago (Wagner et al. 2005).

Some species of Pisonieae have been reported as dominant components of the vegetation from remote islands, in part because their sticky anthocarps can travel long distances attached to the feathers of seabirds (St. John 1951; Airy-Shaw 1952). Therefore, future studies on the taxonomy, ecology and biogeography of Pisonieae will help us understand how interactions with pollinators, seed dispersers (Walker et al. 1991; Murphy and Legge 2003; Burger 2005) and mycorrhiza (Hayward and Hynson 2014) have contributed to promote endemism in trees with a high dispersal capability.

### Taxonomic treatment

#### Ceodes

Taxon classificationPlantaeCaryophyllalesNyctaginaceae

J.R.Forst. & G.Forst., Char. Gen. Pl., ed. 2: 141. 1776.

3CA0F9FD-3ECA-5A00-91C4-D38441DF362E

≡ Pisonia sect. Prismatocarpae Heimerl, Nat. Pflanzenfam. 3(1b): 29. 1889. Type (designated by Stemmerik in Blumea 12: 277. 1964): Pisonia umbellifera (J.R.Forst. & G.Forst.) Seem. (≡ Ceodes umbellifera J.R.Forst. & G.Forst.), **syn. nov.**
= Calpidia Thouars, Hist. Vég. Îsles Austral. Afriq. 37, pl. 10. 1804. Type: Calpidia oblonga J.St.-Hil., **syn. nov.**
= Heimerlia Skottsb., Svensk Bot. Tidskr. 30: 738. 1936 (non Höhn. 1903). Type: Heimerlia brunoniana (Endl.) Skottsb., **syn. nov.**
= Heimerliodendron Skottsb., Svensk Bot. Tidskr. 35: 364. 1941. Type: Heimerliodendron brunonianum (Endl.) Skottsb., **syn. nov.**
= Pisonia sect. Paucistaminatae (Heimerl) Stemm., Blumea 12: 277. 1964 ≡ Calpidia sect. Paucistaminatae Heimerl, Nat. Pflanzenfam. 16c: 125. 1934. Type (designated by Stemmerik in Blumea 12: 277. 1964): Pisonia longirostris Teijsm. & Binn. (≡ Calpidia longirostris (Teijsm. & Binn.) Heimerl), **syn. nov.**
= Pisonia sect. Timeroyea (Montrouz.) Heimerl, Nat. Pflanzenfam. 3(1b): 29. 1889, ‘Timeroya’ ≡ Timeroyea Montrouz., Mém. Acad. Roy. Sci. Lyon, Sect. Sci. 10: 247. 1860. Type (designated by Stemmerik in Blumea 12: 277. 1964): Pisonia artensis (Montrouz.) Barg.-Petr. (≡ Timeroyea artensis Montrouz.), **syn. nov.**
= Vieillardia Brong. & Gris, Bull. Soc. Bot. France 8: 375. 1861 (non Montrouz. 1860). Type: Vieillardia austrocaledonica Brong. & Gris., **syn. nov.**

##### Type.

*C. umbellifera* J.R.Forst. & G.Forst.

##### Description.

***Habit and phyllotaxy*.** Dioecious or hermaphroditic trees or shrubs, leaves (sub)opposite or (sub)verticillate clustered at apex of branches.

***Inflorescence*.** Axillary, terminal or occasionally cauliflorous, arranged in compound cymes.

***Flowers*.** Unisexual (with vestiges of another sex) or rarely hermaphrodite, pedicellate, bracteoles absent at the upper portion of the pedicels, perianth campanulate (Fig. 2A, D) to funnel-shaped, stamens 2 to many (> 30), long or shortly exserted (Fig. 2D) and stigma penicillate or less frequently fimbriate, exserted (Fig. 2A).

***Anthocarp*.** Leathery or woody (but never fleshy), ellipsoid, prismatic or fusiform, sometimes with a rostrum at apex (Fig. 3B) and 4–5 longitudinal ribs with inconspicuous sticky glands (Figs 2B, C, 3A–C).

***Pollen*.** Six and 12 colpi geometrically arranged.

***Perisperm*.** Often scarce, gelatinous or mealy.

#### 1. Ceodes amplifolia

Taxon classificationPlantaeCaryophyllalesNyctaginaceae

(Heimerl) E.F.S.Rossetto & Caraballo comb. nov.

59997B81-33B8-547E-A72B-4D82F227B92D

urn:lsid:ipni.org:names:77210103-1

≡ Ceodes umbellifera f. amplifolia Heimerl, Occas. Pap. Bernice Pauahi Bishop Mus. 13: 38. 1937. (Basionym).

##### Distribution.

French Polynesia (Austral Islands) (Florence 2004).

#### 2. Ceodes artensis

Taxon classificationPlantaeCaryophyllalesNyctaginaceae

(Montrouz.) E.F.S.Rossetto & Caraballo comb. nov.

F44AC0E7-0545-5F07-8F2C-95DE897EFA6D

urn:lsid:ipni.org:names:77210104-1

≡ Timeroyea artensis Montrouz., Mém. Acad. Roy. Sci. Lyon, Sect. Sci. 10: 247. 1860. (Basionym).

##### Distribution.

New Caledonia (Stemmerik 1964a).

#### 3. Ceodes austro-orientalis

Taxon classificationPlantaeCaryophyllalesNyctaginaceae

(J.Florence) E.F.S.Rossetto & Caraballo comb. nov.

F5616801-EA57-52FB-998F-D996B7B46DA6

urn:lsid:ipni.org:names:77210105-1

≡ Pisonia austro-orientalis J. Florence, Fl. Polynésie Franç. 2: 307. 2004. (Basionym).

##### Distribution.

French Polynesia (Gambier Islands) (Florence 2004).

#### 4. Ceodes brownii

Taxon classificationPlantaeCaryophyllalesNyctaginaceae

(J.Florence) E.F.S.Rossetto & Caraballo comb. nov.

9EB49606-9C15-54F5-8EE0-0B1649AB52DE

urn:lsid:ipni.org:names:77210106-1

≡ Pisonia brownii J.Florence, Fl. Polynésie Franç. 2: 308. 2004. (Basionym).

##### Distribution.

French Polynesia: Nuku Hiva (Florence 2004).

#### 5. Ceodes brunoniana

Taxon classificationPlantaeCaryophyllalesNyctaginaceae

(Endl.) Skottsb., Acta Horti Gothob. 2: 231. 1926.

4309BB42-A74E-5D00-9130-02BA9C7BF4BE

Figs 2B , 3A

≡ Pisonia brunoniana Endl., Prodr. Fl. Norf. 43. 1833. (Basionym).

##### Distribution.

Hawai‘i (Hawai‘i, Lana‘i, Maui, Moloka‘i, O‘ahu), Lord Howe and Norfolk Islands and New Zealand (Northern Island) (Heimerl 1913b; Wagner et al. 2005).

##### Note.

*Pisonia brunoniana* Endl., which was considered by Stemmerik (1964a)a synonym of *P. umbellifera*, is treated here as an accepted name (as *Ce. brunoniana*), following Sykes (1987), who clarified the key characters to separate both species.

#### 6. Ceodes cauliflora

Taxon classificationPlantaeCaryophyllalesNyctaginaceae

(Scheff.) E.F.S.Rossetto & Caraballo comb. nov.

7E9E24C3-DEBC-5391-9D46-5A3833639511

urn:lsid:ipni.org:names:77210107-1

≡ Pisonia cauliflora Scheff., Natuurk. Tijdschr. Ned.-Indië 32: 417. 1872. (Basionym).

##### Distribution.

Mariana Islands, Solomon Islands and Lesser Sunda Islands, Moluccas and western Papua New Guinea (Stemmerik 1964b).

#### 7. Ceodes corniculata

Taxon classificationPlantaeCaryophyllalesNyctaginaceae

(Barg.-Petr.) Merr. & L.M.Perry, J. Arnold Arbor. 20: 327. 1939.

1E0F8DBD-1320-5826-A72F-EC9D9E097C8D

≡ Pisonia corniculata Barg.-Petr., Nuov. Giorn. Bot. Ital. ser. 2, 8: 615. 1901. (Basionym).

##### Distribution.

Bacan Islands (Moluccas) and western Papua New Guinea (Heimerl 1913b; Stemmerik 1964b).

#### 8. Ceodes coronata

Taxon classificationPlantaeCaryophyllalesNyctaginaceae

(Heimerl) E.F.S.Rossetto & Caraballo comb. nov.

960EAF81-3C09-5D8C-A0FA-365594F90070

urn:lsid:ipni.org:names:77210108-1

≡ Ceodes umbellifera var. coronata Heimerl, Occas. Pap. Bernice Pauahi Bishop Mus. 13: 41. 1937. (Basionym).

##### Distribution.

Rapa Iti (French Polynesia) (Florence 2004).

#### 9. Ceodes diandra

Taxon classificationPlantaeCaryophyllalesNyctaginaceae

(Pulle) E.F.S.Rossetto & Caraballo comb. nov.

06A84F9F-8740-579F-BF2F-AC3D0129F978

urn:lsid:ipni.org:names:77210110-1

≡ Pisonia diandra Pulle, Nova Guinea 8: 629. 1912. (Basionym).

##### Distribution.

Papua New Guinea (Stemmerik 1964b).

#### 10. Ceodes gigantocarpa

Taxon classificationPlantaeCaryophyllalesNyctaginaceae

(Heimerl) E.F.S.Rossetto & Caraballo comb. nov.

4639DA98-3BD4-5840-84B7-8C16A80CFB6E

urn:lsid:ipni.org:names:77210111-1

≡ Calpidia gigantocarpa Heimerl, Oesterr. Bot. Z. 63: 284. 1913. (Basionym).

##### Distribution.

New Caledonia (Stemmerik 1964a).

#### 11. Ceodes gracilescens

Taxon classificationPlantaeCaryophyllalesNyctaginaceae

(Heimerl) E.F.S.Rossetto & Caraballo comb. nov.

991997D9-A67E-53D6-A16D-5049520A001E

urn:lsid:ipni.org:names:77210112-1

≡ Calpidia gracilescens Heimerl, Oesterr. Bot. Z. 63: 285. 1913. (Basionym).

##### Distribution.

Tahiti (French Polynesia) (Florence 2004).

#### 12. Ceodes lanceolata

Taxon classificationPlantaeCaryophyllalesNyctaginaceae

(Poir.) E.F.S.Rossetto & Caraballo comb. nov.

4FD48785-9145-5CE0-80F2-05C236A2882A

urn:lsid:ipni.org:names:77210113-1

≡ Calpidia lanceolata Poir., Encycl. Suppl. 2: 38. 1811. (Basionym).

##### Distribution.

Mauritius and Réunion islands (Philcox and Coode 1994).

##### Note.

We disagree with Stemmerik’s (1964a) view on *Pisonia lanceolata* (Poir.) Choisy, which he considered a synonym of *P. umbellifera*. According to Friedmann (1986), *P. lanceolata* is a species with affinities to *P. brunoniana*, from which it can be distinguished by the pattern of incisions in the flower.

#### 13. Ceodes longirostris

Taxon classificationPlantaeCaryophyllalesNyctaginaceae

(Teijsm. & Binn.) Merr. & L.M.Perry, J. Arnold Arbor. 20: 328. 1939.

AD3B333D-4849-58F8-A0D3-7E0F9B46EFFB

Fig. 3B

≡ Pisonia longirostris Teijsm. & Binn., Natuurk. Tijdschr. Ned.-Indië 25: 401. 1863. (Basionym).

##### Distribution.

Solomon Islands, Lesser Sunda Islands, Sulu Archipelago (Philippines), Moluccas and Papua New Guinea (Stemmerik 1964b).

#### 14. Ceodes merytifolia

Taxon classificationPlantaeCaryophyllalesNyctaginaceae

(Whistler) E.F.S.Rossetto & Caraballo comb. nov.

0E52E46F-A7EE-5B72-A9C5-B174228B6399

urn:lsid:ipni.org:names:77210114-1

≡ Pisonia merytifolia Whistler, Rainforest Trees Samoa: 192. 2004, ‘*merytafolia*’. (Basionym).

##### Distribution.

Samoa Archipelago (Whistler 2004).

#### 15. Ceodes muelleriana

Taxon classificationPlantaeCaryophyllalesNyctaginaceae

(Warb.) E.F.S.Rossetto & Caraballo comb. nov.

6256C30A-5552-57F2-8F7A-A85D4606C481

urn:lsid:ipni.org:names:77210115-1

≡ Pisonia muelleriana Warb., Bot. Jahrb. Syst. 13: 304. 1891. (Basionym).

##### Distribution.

Solomon Islands and Papua New Guinea (Stemmerik 1964b).

#### 16. Ceodes rapaensis

Taxon classificationPlantaeCaryophyllalesNyctaginaceae

(J.Florence) E.F.S.Rossetto & Caraballo comb. nov.

723FB57F-9527-5B92-9D21-2D14828B70A9

urn:lsid:ipni.org:names:77210116-1

≡ Pisonia rapaensis J.Florence, Fl. Polynésie Franç. 2: 317. 2004. (Basionym).

##### Distribution.

French Polynesia (Rapa Iti) (Florence 2004).

#### 17. Ceodes sechellarum

Taxon classificationPlantaeCaryophyllalesNyctaginaceae

(F.Friedmann) E.F.S.Rossetto & Caraballo comb. nov.

0B878D62-93C1-5125-97C8-55F186F7541C

urn:lsid:ipni.org:names:77210117-1

≡ Pisonia sechellarum F.Friedmann, Bull. Mus. Natl. Hist. Nat., B, Adansonia, sér. 4, 8: 384. 1986 (publ. 1987). (Basionym).

##### Distribution.

Seychelles (Silhouette Island) (Friedmann 1986).

#### 18. Ceodes taitensis

Taxon classificationPlantaeCaryophyllalesNyctaginaceae

(Heimerl) E.F.S.Rossetto & Caraballo comb. nov.

C6B0DC1E-02DC-5E31-95D9-83F67567688D

urn:lsid:ipni.org:names:77210118-1

Fig. 2A

≡ Calpidia taitensis Heimerl, Oesterr. Bot. Z. 63: 288. 1913. (Basionym).

##### Distribution.

French Polynesia (Society Islands) (Florence 2004).

#### 19. Ceodes umbellifera

Taxon classificationPlantaeCaryophyllalesNyctaginaceae

J.R.Forst & G.Forst., Char. Gen. Pl., ed. 2 142, t. 71. 1776.

90A68208-BE1A-5FEF-8662-B3FC1973124D

Figs 2C, D , 3C

##### Distribution.

Widespread across the Indo-Pacific islands (Pramanick et al. 2015).

#### 20. Ceodes wagneriana

Taxon classificationPlantaeCaryophyllalesNyctaginaceae

(Fosberg) E.F.S.Rossetto & Caraballo comb. nov.

4D3EBE3E-D459-5EC9-8303-DDA1AE15511D

urn:lsid:ipni.org:names:77210119-1

≡ Pisonia wagneriana Fosberg, Phytologia 62: 177. 1987. (Basionym).

##### Distribution.

Hawai‘i (Kaua‘i) (Fosberg 1987).

#### Rockia

Taxon classificationPlantaeCaryophyllalesNyctaginaceae

Heimerl, Oesterr. Bot. Z. 63: 289. 1913.

C19ECE5C-24D3-5862-9B2C-4D265FAA9A34

##### Type.

*R. sandwicensis* (Hillebr.) Heimerl.

##### Description.

***Habit and phyllotaxy*.** Dioecious trees or shrubs, leaves (sub)opposite or (sub)verticillate clustered at apex of branches.

***Inflorescence*.** Axillary, terminal, arranged in compound cymes.

***Flowers*.** Unisexual (with vestiges of another sex), sessile, with one bract and two bracteoles present at the base, male perianth campanulate, stamens 10–26, exserted (Fig. 2G), female perianth tubular or fusiform (Fig. 2H), stigma fimbriate, exserted.

***Anthocarp*.** Leathery, elongated fusiform, with 5 ribs covered by inconspicuous glands excreting sticky substances (Figs 2I, 3F).

***Pollen.*** Tricolpate, with 3 apertures distant 120° from each other.

***Perisperm*.** Abundant, gelatinous.

#### 1. Rockia sandwicensis

Taxon classificationPlantaeCaryophyllalesNyctaginaceae

(Hillebr.) Heimerl, Oesterr. Bot. Z. 63: 290. 1913.

6C8DB932-667C-5D7F-92C8-99FCC40837F5

Figs 2G–I , 3F

≡ Pisonia sandwicensis Hillebr., Fl. Hawaiian Isl. 369. 1888. (Basionym).

##### Distribution.

Hawai‘i (Hawai‘i, Kaua‘i, Lana‘i, Maui, Moloka‘i, O‘ahu) (Stemmerik 1964a; Wagner et al. 2005).

### Key to genera from tribe Pisonieae

The following dichotomous key is compiled to separate the nine currently accepted genera within Pisonieae, based on reproductive features because vegetative (i.e. leaves and twigs) characters do not seem to provide enough resolution to help set apart these genera. Unfortunately, members of Pisonieae tend to have fugacious reproductive seasons and many collections in herbaria lack flowers and/or fruits. Thus, we support the recommendation made by Caraballo-Ortiz and Trejo-Torres (2017) on preparing multiple vouchers from a plant or population across seasons to document the full phenology of species and their range of morphological variation.

**Table:** 

1	Staminate flowers with inserted stamens	**2**
–	Staminate flowers with exserted stamens	**4**
2	Stamens about 30	*** Cephalotomandra ***
–	Stamens 5–13	**3**
3	Leaves drying blackish; inflorescences in corymbose cymes; flowers usually with urceolate corolla. Widespread across the Neotropics	*** Neea ***
–	Leaves greenish when dry; inflorescences in dichasium; flowers with campanulate corolla. Restricted to Guatemala	*** Neeopsis ***
4	Flowers pedicellate, lacking bracts or bracteoles at the upper portion of the pedicels	**5**
–	Flowers sessile, subtended by one bract and two bracteoles	**6**
5	Inflorescence in simple umbel; glandular emergences along anthocarp ribs. Neotropics	*** Pisoniella ***
–	Inflorescence in compound cymes; inconspicuous glands along anthocarp ribs. Indo-Pacific	*** Ceodes ***
6	Anthocarps red- or violet coloured, more or less fleshy when ripe	*** Guapira ***
–	Anthocarps dry when ripe	**7**
7	Anthocarps winged, lacking sticky glands	*** Grajalesia ***
–	Anthocarps not winged, sticky glands present	**8**
8	Anthocarp ribs covered by glandular emergences. Pantropical	*** Pisonia ***
–	Anthocarp ribs covered by inconspicuous glands. Endemic to Hawai‘i	*** Rockia ***

## Supplementary Material

XML Treatment for Ceodes

XML Treatment for Ceodes amplifolia

XML Treatment for Ceodes artensis

XML Treatment for Ceodes austro-orientalis

XML Treatment for Ceodes brownii

XML Treatment for Ceodes brunoniana

XML Treatment for Ceodes cauliflora

XML Treatment for Ceodes corniculata

XML Treatment for Ceodes coronata

XML Treatment for Ceodes diandra

XML Treatment for Ceodes gigantocarpa

XML Treatment for Ceodes gracilescens

XML Treatment for Ceodes lanceolata

XML Treatment for Ceodes longirostris

XML Treatment for Ceodes merytifolia

XML Treatment for Ceodes muelleriana

XML Treatment for Ceodes rapaensis

XML Treatment for Ceodes sechellarum

XML Treatment for Ceodes taitensis

XML Treatment for Ceodes umbellifera

XML Treatment for Ceodes wagneriana

XML Treatment for Rockia

XML Treatment for Rockia sandwicensis
